# Ten simple rules for annotating sequencing experiments

**DOI:** 10.1371/journal.pcbi.1008260

**Published:** 2020-10-05

**Authors:** Irene Stevens, Abdul Kadir Mukarram, Matthias Hörtenhuber, Terrence F. Meehan, Johan Rung, Carsten O. Daub

**Affiliations:** 1 Department of Biosciences and Nutrition, Karolinska Institutet, Huddinge, Sweden; 2 Science for Life Laboratory, Karolinska Institutet, Stockholm, Sweden; 3 Department of Immunology, Genetics and Pathology, Uppsala University, Uppsala, Sweden; 4 European Molecular Biology Laboratory, European Bioinformatics Institute, Wellcome Trust Genome Campus, Hinxton, Cambridge, United Kingdom; Dassault Systemes BIOVIA, UNITED STATES

## Introduction

A file of nucleic acid sequences itself is not descriptive. Accompanying information describing data, known as metadata, is important for fueling artificial intelligence and ensuring data longevity as technologies evolve. Poor metadata can significantly lower the value of sequencing experiments by limiting the reproducibility of the study and its reuse in integrative analyses. Furthermore, metadata provides the basis for supervised machine learning algorithms using labeled data and indexing Next Generation Sequencing datasets into public repositories to support database queries and data discovery. Thus, metadata is key for making data Findable, Accessible, Interoperable, and Reusable (FAIR) [1].

Several empirical studies have shown the need for better practices in curating scientific data [2–5]. Community efforts to improve metadata quality include various minimum metadata standards such as Minimum Information about a Next-Generation Sequencing Experiment (MINSEQE) [6] or broader principles such as the FAIR guidelines. However, there is a lack of consensus or compliance for many of these standards.

**Fig 1:**
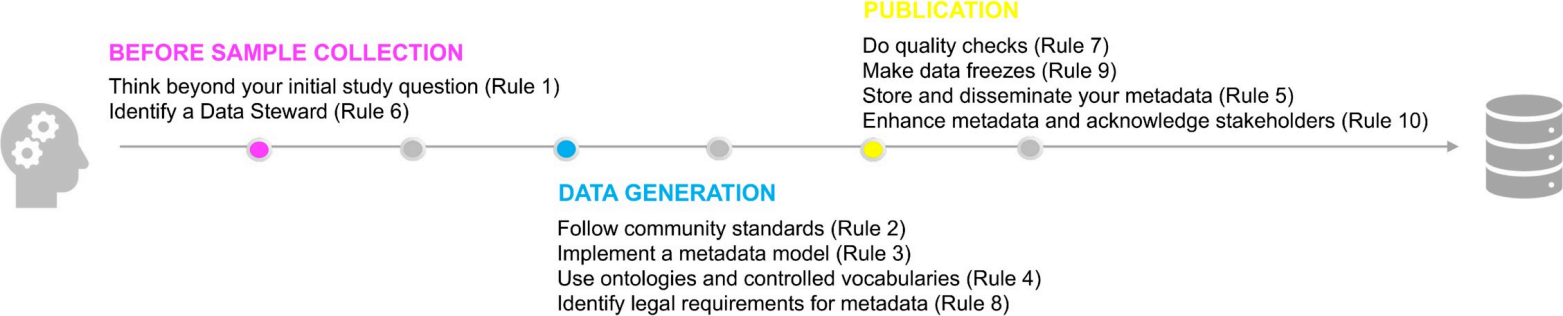
Summary of recommendations for metadata collection at 3 key stages of a sequencing project: before samples collection, during data production, and prior to publication. Note that the rules become increasingly more concrete as the project progresses.

Here, we distilled a few pragmatic principles, which are summarized in Fig 1, to help data producers collect and store high-quality metadata about sequencing experiments. Ultimately, we hope these will increase the resource value of public sequencing data.

## Rule 1: Think beyond your initial study question

Metadata is usually specific to a given study, thus the decision of what metadata to collect should be largely determined during the experimental design phase knowing what variables will be created. Think beyond your immediate biological questions, and record everything that systematically varies in the experiment. As early as sample collection, record sufficient descriptive information that will allow others to reproduce your experiment. After sample collection is finished, it will be more difficult to remember sample details, for example, since key personnel might not be present anymore in the lab. Remember to add sufficient details needed to reproduce your study or to support database queries that will discover your data. An example of something which might be missed is information about DNA or RNA fragmentation, sequencing adapter ligation, and library enrichment steps prior to sequencing. Alnasir and colleagues [7] report only 4% of metadata records in the MINSEQE-compliant Sequence Read Archive (SRA) repository contain information about these protocol steps, causing biases in meta-analyses of SRA records.

In addition to experimental details, the metadata record should also provide technical details such as barcodes, linkers, and other nucleotide information. Capture the computational aspects such as processing pipelines and the respective software versions. Publish your code and processing environment as a Git repository, Docker container, computational notebook, or Code Ocean capsule. Provide all the code and data needed to reproduce your figures (e.g., count tables). In subsequent rules, we give progressively more concrete ways to design (see Rule 2) and implement (see Rule 3) custom metadata records.

## Rule 2: Follow community standards

Meta-analyses, increasingly performed using machine learning approaches, are using metadata to incorporate disparate datasets and find new insights into biological processes. To ensure compatibility of your study with similar studies, adhere to established community standards and formats for metadata and data.

The FAIR guidelines [1] offer high-level advice for making data FAIR. The MINSEQE standard [8] was established by the Functional Genomics Data Society (FGED) similar to the Minimum Information About a Microarray Experiment (MIAME) standard for microarrays [9]. These standards are intended to provide the minimum descriptive information to enable data reuse, and many public repositories are MINSEQE compliant. The Dublin Core Metadata Initiative [10] developed standards and best practice recommendations for creating and sharing metadata, available through the Dublin Core User Guide (dublincore.org/resources/userguide/). The Global Alliance for Genomics and Health (GA4GH) [11] also provides standards and tools for sequencing data, such as the Genomic Data Toolkit (ga4gh.org/genomic-data-toolkit/).

As a first step, determine the minimum standards and requirements of your target repository and journal. Adhering to these requirements is a prerequisite for publishing scientific data. Beyond the minimum standards, it is strongly encouraged to add as much experimental detail as possible.

## Rule 3: Implement a metadata model

A metadata model spells out the terms, relationships, and categories used to describe samples and data in a structured manner. One example of a metadata model is the International Human Epigenome Consortium (IHEC) metadata model [12]. Several large-scale sequencing projects, such as the Functional Annotation of the Mammalian Genome (FANTOM5) [13], Encyclopedia of DNA Elements (ENCODE) [14], and the Danio Rerio Encyclopedia of DNA Elements (DANIO-CODE) [15], have established additional metadata models to customarily describe their data in a systematic way that allows for integrative analysis of disparate datasets.

Create a similar metadata specification by listing all the possible terms that will describe your data. Organize terms into progressively broader categories until obtaining only a few umbrella categories that reflect the experimental workflow from sample collection to data processing. Within each category, providing certain terms may be required or optional based on how these are used in downstream analysis.

We previously created a custom metadata specification using a similar approach [16]. We used a top-down structure to capture metadata across the entire experimental workflow from biological sample to library preparation, sequencing procedure, sequencing files, and processed files. We defined 6 metadata sections corresponding to the experiment workflow: Series, Biosample, Assay, Applied Assay, Sequencing, and Data. Under each section, we defined weights on the terms such as required (e.g., biosample type), conditionally required (e.g., target of a chromatin immunoprecipitation sequencing (ChIP-seq assay)), and optional terms (e.g., chemistry version used for sequencing).

The Investigation/Study/Assay Tab-Delimited (ISA-TAB) [17] format is widely used for submitting metadata to repositories. The ISA-TAB format can be implemented as text-based, such as comma-separated values (CSV), tab-separated values (TSV), Excel-based, or relational database depending on the data volume and project resources.

For a smaller sequencing project, it might be useful to take advantage of tools specifically designed for capturing metadata, such as the Center for Expanded Data Annotation and Retrieval (CEDAR) Workbench [18] or ISA-TAB tools [19] (isa-tools.org/index.html). For larger projects, custom implementations can be considered such as the ENCODE Data Coordination Center (DCC) [14] or FANTOM5 Semantic catalogue of Samples, Transcription Initiation, And Regulations (SSTAR) [13].

To help mitigate potential reproducibility issues, consider using workflow management tools (e.g., nf-core [20], Cromwell [21], and Galaxy [22]) and workflow description standards (Common Workflow Language (CWL) [23] and Workflow Description Language (WDL) [21]).

## Rule 4: Use ontologies and controlled vocabularies

Maximize the use of ontologies and controlled vocabularies within the metadata fields (see Rule 3). This will reduce misannotations and ensure metadata consistency and compatibility with other datasets. We recommend using a minimum set of ontologies to describe samples (i.e., cell lines, primary cells, and primary tissues), sequencing details (assay types and platforms), or diseases. Useful resources are the Open Biological and Biomedical Ontology (OBO) Foundry [24], National Center for Biomedical Ontology (NCBO) BioPortal [25], or European Bioinformatics Institute (EBI) Ontology Lookup service [26].

When an ontology is not available, consider using controlled vocabulary terms to minimize misannotations in the metadata. For example, create a list of controlled terms such as for file formats (e.g., FASTQ and BAM), for sequencing instruments (e.g., HiSeq X, etc.), or for platforms (Illumina, Ion Torrent, PacBio, etc.) in order to restrict entries to a predefined vocabulary. This will limit the introduction of errors in the metadata record and ease the data input as well.

## Rule 5: Store and disseminate your metadata

It is best practice to create a data management plan (DMP) before generating research data [27]. One component of any DMP is the infrastructure for delivery, analysis, and long-term storage of sequencing data and its description. Give careful consideration to the security, data loss prevention, and ease of accessibility for collaborators and analysts. Any metadata that contains potentially sensitive information should be encrypted and stored in a secure location. Data loss prevention includes measures such as automated backups, storage in multiple locations, and long-term archiving considerations. Metadata should still be easy to share with the research community and collaborators.

Several publicly funded resources are available for long-term archiving and dissemination of sequencing data and accompanying metadata. The National Center for Biotechnology Information database of Genotypes and Phenotypes (NCBI dbGAP) [28] and the European Genome-phenome Archive (EGA) [29] resources specialize in permanent archiving and sharing of personally identifiable genetic and phenotypic data resulting from biomedical research projects including sequencing data. For data that are not personally identifiable, the NCBI SRA [30], the European Nucleotide Archive (ENA) [31], and the DNA Databank of Japan [32] make biological sequence data available to the research community. GEO [33] and BioSamples [34] collect mainly metadata and references to the respective sequencing data in other databases. In addition, institutional repositories (IRs) funded by the host institution may provide additional storage and data dissemination mechanisms as a complement to specialized public sequence repositories. Some examples of IRs are the Science for Life Lab Data Centre (www.scilifelab.se/data/) and the Beijing Institute of Genomics (BIG) Data Center [35].

Consider data and metadata submission requirements when developing a DMP. In case you propose a large-scale project, consider reaching out for input to streamline future submissions.

## Rule 6: Identify a data steward

The data production process spans several stages. Thus, metadata collected over an extended time span might not always be complete or consistent. Sometimes, key personnel move on, causing projects to fail moving forward. The best practice is to assign 1 person from the beginning of the project to be responsible for maintaining and periodically reviewing data records. It can be a data manager, a data officer, or any person with data management competence. Ensure this person will stay engaged throughout the life span of the project. This will allow them to identify issues before key personnel move on to other projects. The data steward can also ensure that policy decisions are applied consistently and timely.

Some institutions provide data support, such as information about data policy, help with making DMPs, or e-infrastructure resources. Take advantage of the data resources provided by your institution and ensure compliance with university policies.

## Rule 7: Do quality checks

Quality control of sequencing data is important, but it is beyond the scope of this paper. Here, we focus on metadata quality checks as rapid ways to identify inconsistencies and eliminate errors in the metadata. Perform checks systematically as early as the sample collection phase. Beyond that, validate the accuracy of the metadata against the data. For example, a sample is supposed to be male or female, or a certain gene should be knocked out in the sample. More detailed validations can use data-driven methods, such as clustering samples and identifying outliers. Identify and flag missing values, validate entries against accepted ontology or controlled terms, and validate file formats. Avoid recording 0 for missing values, rather use an appropriate flag (e.g., NA). We recommend designing a file naming scheme and discarding poor quality data early to avoid duplication of records. Be clear about the meaning of terms used in describing your data. For example, clearly distinguish technical and biological replicates. Finally, ask the data generator to verify their metadata. Manual curation remains the gold standard for ensuring high-quality metadata.

## Rule 8: Identify legal requirements for metadata

Sequencing experiments in human samples raise special ethical and regulatory concerns. The principal investigator is responsible to be aware of and comply with national or regional legal policies applicable to the location where the data are physically stored. Sensitive metadata likewise must comply with domestic and international standards, including the General Data Protection Regulation (GDPR) and Health Insurance Portability and Accountability Act (HIPAA). Verify the requirements of the funding agency, publishing journal, or university for sensitive data. For medical grade sequencing data, additional standards exist, such as ISO13485:2016 or ISO 27001.

## Rule 9: Make data freezes

Data changes with time as files are reprocessed, and metadata is corrected or added. A data freeze is a snapshot of raw and processed sequence files, metadata, and computational workflows at specific time points. Large consortium projects such as FANTOM5 [13] and ENCODE [12] manage ever evolving datasets and metadata by performing periodic data freezes. However, any sequencing project, whether large or small, can benefit from freezing data by creating a resource that will never be changed and can be referenced later on. Each freeze captures the state of data in a system that can be used as a reference point for future analyses.

Match major updates throughout the life span of your project by data freezes. In the best case, a freeze documentation (User’s Manual) with the version number and time-stamped changelog is created alongside every freeze. Importantly, no modifications may be done to a data freeze, and any changes have to be realized by additional data freezes.

## Rule 10: Enhance metadata and acknowledge stakeholders

Enable people to find your data and quickly get an overview before inspecting the metadata spreadsheets or flat files by giving a graphical abstract, summary statistics on data (dataset size, etc.), or provide a track hub for genome browsers.

Finally, the metadata record is a good place to acknowledge contributors to your data, for example, sequencing centers, data centers, funding agencies, etc. Make sure to use the correct identifiers provided by the funding agencies (project grant numbers) or sequencing centers. This will allow research institutions and funding bodies who are parsing metadata to generate summary metrics about the scientific output and impact of the work. It will also ensure continued backing for your institution’s support departments.

## Conclusion

As sequencing technologies evolve, investigators generate an increasing amount of genomics data. Each sequencing sample may be described by many aspects (metadata) including experimental details, sequencing protocol, and computational steps. This description is directly linked to the longevity and future reuse of sequencing datasets. Here, we distilled some advice on how to address the challenges of high-quality metadata collection for research groups without dedicated data support.
